# Prediction of Local Quality of Protein Structure Models Considering Spatial Neighbors in Graphical Models

**DOI:** 10.1038/srep40629

**Published:** 2017-01-11

**Authors:** Woong-Hee Shin, Xuejiao Kang, Jian Zhang, Daisuke Kihara

**Affiliations:** 1 Department of Biological Science, Purdue University, 249 S. Martin Jischke Street, West Lafayette, IN, USA; 2 Department of Computer Science, Purdue University, 305 N. University Street, West Lafayette, IN, USA

## Abstract

Protein tertiary structure prediction methods have matured in recent years. However, some proteins defy accurate prediction due to factors such as inadequate template structures. While existing model quality assessment methods predict global model quality relatively well, there is substantial room for improvement in local quality assessment, i.e. assessment of the error at each residue position in a model. Local quality is a very important information for practical applications of structure models such as interpreting/designing site-directed mutagenesis of proteins. We have developed a novel local quality assessment method for protein tertiary structure models. The method, named Graph-based Model Quality assessment method (GMQ), explicitly considers the predicted quality of spatially neighboring residues using a graph representation of a query protein structure model. GMQ uses conditional random field as its core of the algorithm, and performs a binary prediction of the quality of each residue in a model, indicating if a residue position is likely to be within an error cutoff or not. The accuracy of GMQ was improved by considering larger graphs to include quality information of more surrounding residues. Moreover, we found that using different edge weights in graphs reflecting different secondary structures further improves the accuracy. GMQ showed competitive performance on a benchmark for quality assessment of structure models from the Critical Assessment of Techniques for Protein Structure Prediction (CASP).

Protein structure prediction methods have steadily improved over the past two decades, with a rise in the availability of software and more routine use of computational models in biological studies^1^. However, constructing an accurate model is still not always possible even with state-of-the-art methods^2^,^3^. The accuracy of a structure model depends on various aspects including the availability of solved structures of related proteins that can be used as modeling templates. Because the accuracy of models varies, knowing the accuracy of a specific computational model is crucial for practical use of that model for biological studies. Predicting the accuracy of protein tertiary structure models is known as model quality assessment (MQA) and has become an active research topic in structural bioinformatics^4^.

MQA is not just for identifying highly accurate models. Models of only moderate accuracy are nonetheless useful for many purposes^5^. Models of an atomic-detailed accuracy with a root-mean-square deviation (RMSD) of 1.5–2 Å to the native structure are useful for almost any application where structure information is beneficial, such as studying enzymatic mechanism and protein engineering, and drug design^6^. Models with a correct backbone orientation (e.g. an RSMD of 4 to 6 Å) can be used for applications that need residue position level accuracy, e.g. designing and interpreting site-directed mutagenesis experiments. Models with a slightly higher (worse) RMSD but nearly correct overall fold may be used for predicting function from their global fold^7^, identifying local functional sites^8^,^9^,^10^,^11^, or for interpretation of low-resolution structural data^12^. Appropriate use of a model for the applications listed above is only possible when users know the accuracy of the model. Therefore, it is crucial to establish methods that assess the quality of protein structure models so that they can be used in applications suitable for their estimated accuracy. MQA is also an indispensable step for refining structure models^13^.

There are two classes of protein MQA methods that predict, respectively, global and local quality of a structure model. The former class of methods predicts RMSD or other related metrics that indicate global structural similarity of a model to the protein native structure. By comparison, local quality prediction methods aim to indicate the accuracy or error at each residue of a model, e.g. the distance between the position of Cα atoms in the predicted and native structures. More QA methods have been developed for predicting global rather than local quality. Moreover, the former have been more successful as reported in a community-wide protein structure prediction experiments, Critical Assessment of Techniques for Protein Structure Prediction (CASP)^4^. Global quality of models can be predicted from structural and sequence (target-template alignment) features of models or combinations thereof, often formulated in a machine learning framework. Useful structural features include residue/atom-contact potentials^14^,^15^, main-chain torsion angles^16^, burial/exposure propensity of residues, and residue environment propensity^17^, while alignment features include alignment scores computed for a target and a template sequence/profile the sequence identity, and their statistical significance^18^,^19^. To combine features, methods such as linear combination^20^ or regression^21^, support vector machine (SVM)^22^,^23^, and neural network^24^,^25^,^26^ have been used. In CASP, consensus approaches, which examine the consistency of models built by different structure prediction methods, performed well^23^,^27^,^28^. For more details about recent trends of MQA methods, refer to a review article^29^.

In contrast to global QA methods, local QA methods need large improvements to be useful in practice. It was reported that the correlation coefficients of predicted and actual local errors in structure models are significantly lower than that of global quality prediction^4^. Local quality information tells users regions or residues in a structure model that are accurately predicted. Thus, it provides very important information for practical application of structural models. Existing local QA methods take similar approaches to global QA methods: structural and sequence-based features of residues in a model are considered in a machine learning framework, e.g. neural networks^30^ and SVM^31^. Other recently developed local QA methods include SMOQ^32^, MULTICOM^33^,^34^,^35^, and Wang_deep^36^.

Here we have developed a novel method for predicting local QA of protein structure models. This new method, named Graph-based Model Quality assessment method (GMQ), uses a new approach in representing a protein model that takes into account the neighboring environment of residues. In GMQ, a target structure model is represented as a graph where surrounding residues that are physically closer than a certain cutoff distance to a residue of interest are connected by edges. Then, for predicting a local quality value (the Euclidean distance between the predicted and the actual Cα position of residues), features of the target residue as well as the predicted quality of neighboring residues are taken into account. This is achieved by using a probabilistic graphical model named Conditional Random Field (CRF)^37^. We show that the accuracy of local quality improves as we consider the predicted quality of residues distant from the target residue. Taking quality of surrounding residues into account is intuitive because it is natural to speculate that the accuracy of the predicted position of a residue is influenced by that of contacting residues. This is a clear difference with existing methods, which use features of only the target residue or consider features of residues that are sequence neighbors but consider neither structural neighbors nor the predicted accuracy of neighbors.

Since the primary aim of GMQ is to provide quality assessment of a computational structure model for biologists who want to use the model, GMQ predicts the real-value of errors (i.e. the range of real-value errors in Angstrom) of a single model rather than ranking many different models. The latter type of QA methods is useful in specific occasions, e.g. CASP, where participants of this prediction assessment need to choose most accurate models among hundreds of models^38^,^39^, but may not be very useful in actual research scenarios where error of a single model is in question. We benchmarked GMQ on a large dataset of structure models of various accuracies as well as structure models of CASP targets.

## Methods

### Graphical Representation of Protein Structure Models

**Figure 1:**
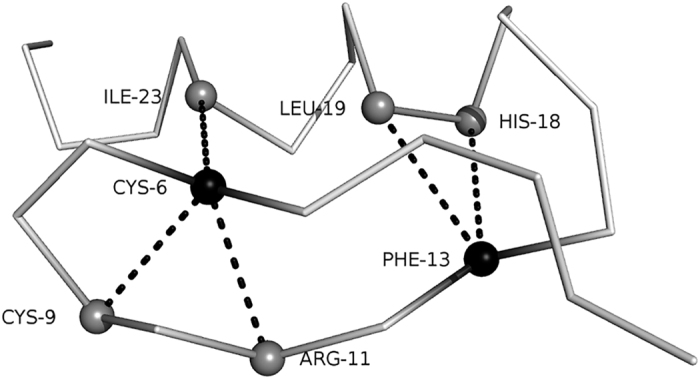
An example of neighboring residues. They are connected with edges in a graph. This is a prediction model for Protein Data Bank (PDB) entry 1AAY with an RMSD of 0.66 Å. 5.5 Å was used for the neighbor cutoff distance.

A protein structure model to be evaluated was represented by a graph where nodes represent Cα positions of residues in the model. Edges were drawn between adjacent residues on the main-chain as well as residues closer than a distance cutoff value. As a distance cutoff, we used 4.0, 4.5, 5.0, and 5.5 Å between residues. 4.0 Å was chosen as the smallest cutoff to include adjacent residues on sequence, considering that the average Cα–Cα distance in native protein structures is 3.8 Å. The largest cutoff value used was 5.5 Å, because a larger cutoff made large cliques in a protein model, which made the computational time significantly longer to complete. In the example shown in Fig. 1, residues that are distant on the sequence but close in the three-dimensional space are connected by edges: residue 6 is connected to 9, 11, and 23, while residue 13 is connected to 18 and 19. Furthermore, we connected residues with a shared neighbor (e.g. ILE23 and CYS9, ILE23 and ARG11).

Prediction of the quality of residues in a structure model was performed for sub-graphs called cliques as units. A clique is a fully connected sub-graph in the entire graph. Thus, a protein structure model is represented by a set of cliques. Neighboring cliques overlap, i.e. some nodes can belong to both of the neighboring cliques. Adjacent cliques are recorded as a tree (clique-tree).

We constructed a CRF for predicting local QA of residues in a protein graph. A CRF is a graphical model for computing the probability of a possible output given the input which is also called the observation^40^. In this work, the output is a vector of the binary classification (labels) that indicates the accuracy of predicted Cα positions in a model, either within or larger than a certain error (Å). The input is features of residues in the model. The probability of an output label (vector) *Y* given input variables (vector) *X* is given as


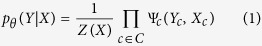


where *θ* is a set of parameters of the model, function Ψ_*c*_ is called factors, *c* is a clique, *C* is a set of all cliques in the graph, *Y*_*c*_, is a set of labels in *c, X*_*c*_ is a set of features for nodes in *c. Z(x*) is a normalization factor:


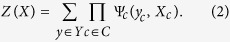


The factor Ψ_*c*_ has a potential function *F* that combines different features of the nodes (amino acid residues) in the clique *c*:


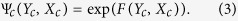


*F* has two components, a term for taking features of the target residue into account and another one that considers predicted labels of neighboring residues:


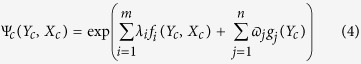


The first term concerns local features of each residue while the second term is for considering predicted labels of neighboring residues in each clique. Intuitively, the first term tries to predict the label of a residue (i.e. if a residue position in the model is within an error cutoff or not) from features of the residue. *m* is the number of features that characterize each residue (*m*  =  25 in this study). The second term considers combinations of labels of neighboring residues. The prediction of a residue is affected by the predicted labels of its neighboring residues. For example, if the quality of neighboring residues are all good, it is more likely that the quality of the residue in the middle is also good. *n* is the number of combinations of binary labels in neighboring residue pairs. Since each residue is classified either below or above a cutoff error, for a connected residue pair, there are *n*  =  4 patterns of binary label combinations, i.e. 00, 01, 10, and 11, where 0 denotes that the residue is predicted to be above the cutoff error while 1 indicates that the residue is predicted to be below the cutoff error, i.e. good quality. *g*_*j*_ indicates the number of times that the corresponding label combinations, 00, 01, 10, and 11, appear in the clique, and ω_j_ is the weight for that combination. For example, for a clique of three residues, A, B, and C, there are three connections, A-B, A-C, and B-C, which results in 2^3^  =  8 combinations of possible label patterns of the three residues, i.e. 000, 001, 010, 100, 011, 101, 110, and 111. For each of these eight label patterns, a score is computed by counting the number of times 00, 01, 10, and 11 occurred in the three connections, and sum of the weight ω_j_ for each pair pattern is computed.

The training of the parameters λ and ω was performed with the stochastic gradient descent method^41^. Local quality prediction corresponds to finding the most likely sequence of labels *Y* for given observed features *X*. The message passing algorithm is used for this task. In short, it is an iterative optimization process where labels optimized in a clique are propagated to the adjacent cliques according to the constructed clique tree to update labels of the entire graph.

### Residue Features

Residues in a protein structure model were characterized by a total of 25 structural and alignment features. We used the same set of features as our previous work^19^,^21^. They can be classified into structural, target-template alignment-based, and composite scores. The structural features include (1) scores that evaluate atoms and residue contacts: ANOLEA1, the percentage of residues with a high energy in a model; ANOLEA2, a non-local normalized energy^42^, Discrete Optimized Protein Energy (DOPE)^14^; (2) a score for torsion angles: TAP^16^; (3) an atomic-detail stereochemical feature from PROCHECK, the percentage of residues that fall in the disallowed region in the Ramachandran map^43^; and (4) four different residue environment scores derived from Verify3D^17^,^44^, i.e. the local Verify3D score assigned to each residue, the global Verify3D score, which is an attribute of a whole model; Verify3D/L^2^, Verify3D score normalized by square of the length, log(Verify3D/L^2^).

Alignment-features used were the amino acid similarity score, which is the average BLOSUM45 score^45^ of columns in the multiple sequence alignment (MSA); the gap ratio, which is the fraction of gaps in columns in the MSA; conservation, the fraction of the most abundant residue type at columns; PRSS^46^, which is a Z-score of the target-template alignment score computed against a distribution of the alignment score of shuffled sequences; and four scores derived from SPAD^19^, which quantifies the consistency of a template-target alignment with a set of suboptimal alignments: local SPAD, which is the SPAD score computed for each residue; global SPAD, which is the average local SPAD (lSPAD) along the entire model; log(SPAD), and log(lSPAD/(SPAD + 1)).

In addition, two global features of models were also considered: the contact order of structures^47^ and its length-normalized version, the length (i.e. the number of amino acids) of the model, and the length of aligned regions of the target protein to its template structure.

Finally, four composite scores were incorporated: GA341^48^, which combines residue-level statistical potentials, the target–template sequence identity, and a measure of structural compactness; pG^48^, which is a probability that a model is correct computed using a Bayesian classifier with the GA341 score and the protein length of the model; and two scores from a neural network-based QA method, ProQ^24^, which were trained for predicting MaxSub or LG score (global structural similarity of models to the native structure). For more details of each score, please refer to our previous paper^21^. Since the ranges of values of these features vary, the values were recast into Z-scores considering the value distribution in the training set.

Among the 25 features, six are local features that are attributed to each residue while the rest are global features assigned to each model (thus residues in the same model have the same value). The local features are local Verify3D score, mutation score, gap ratio, residue conservation, local SPAD, and log(lSPAD/(SPAD + 1)). In addition to a CRF that uses all 25 features, we have also made predictions using only five features, local Verify3D, log(SPAD), log(Verify3D/L^2^), gap ratio, and log(localSPAD/(SPAD + 1)). These features are selected in our previous work of the Sub-AQUA quality assessment method^21^ for local quality by a forward selection procedure for regression analysis.

The program GMQ is made available for the academic community at http://kiharalab.org/gmq.

### Benchmark Datasets

We used two benchmark datasets. The first set contains 1386 template-based models constructed using the Lindahl & Elofsson dataset (L–E dataset)^49^. This data set consists of 1130 representative proteins, each of which is assigned with a SCOP hierarchical structure classification of a family, a superfamily, and a fold^50^. Following the classification, pairs of proteins were selected that belong to the same family, pairs that belong to the same superfamily but not in the same family, and those which belong to the same fold but not in the same superfamily. For a protein pair, one protein was considered as the target protein to be modeled and another one was considered as a template. A pairwise alignment of the two proteins was constructed by a profile-based threading algorithm, SUPRB^51^. Then, using the alignments, a tertiary structure model of the target protein was constructed by a homology modeling software, Modeller^52^ with the default parameter setting. In this study, we used 584 models from the family-level alignments, 307 from the superfamily-level, and 495 fold-level models. The RMSD of the models ranged from 0.36 to 9.99 Å. The structure models contained in total of 159,937 residues.

The second dataset was structure models available from the websites of CASP9, CASP10 and CASP11, which were submitted by participants of the CASP contests. For the CASP9 benchmark, we chose the first model by each structure prediction group for each target. We removed targets from our dataset if the number of models (i.e. the groups submitting their models) was less than 50. This resulted in 5836 structure models from 70 groups for 115 targets. For the latest two CASP benchmark datasets, we downloaded all the models of Stage 2, and removed targets that were cancelled and PDB structures were not available. This led 13,500 models from 90 targets and 10,200 models from 68 targets for CASP10 and CASP11, respectively.

### Performance Evaluation

Since CRF is a binary classifier, we primarily evaluated the quality assessment performance of GMQ by the percentage of residues whose label (whether the error of a residue is smaller or larger than a cutoff distance) was correctly predicted. As a cutoff distance for the definition of correct prediction, we used 2.0, 4.0, 5.0, 6.0, and 8.0 Å between predicted and actual Cα atom positions computed after the global superimposition of a model to the native structure of the protein.

**Figure 2:**
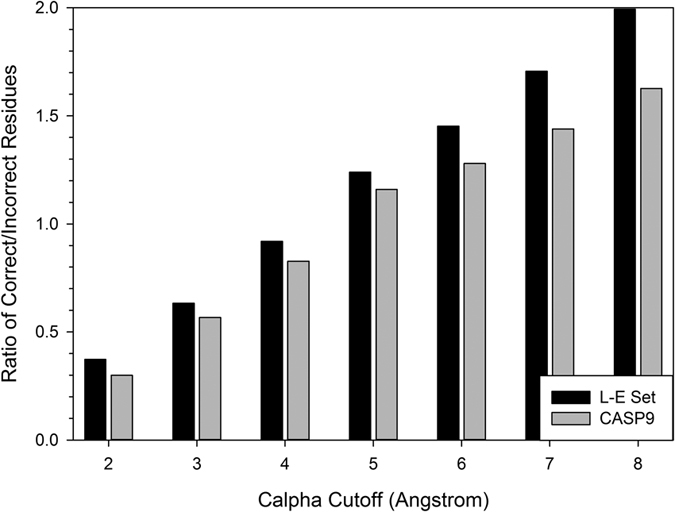
Ratio of correct and incorrect residues under different *Cα* distance cutoffs in the benchmark datasets. For CASP models, those from CASP9 were used. A residue position in a computational structure model is considered to be correct if the distance between the predicted and the actual position of the Cα atom is below a cutoff value.

Figure 2 shows the distribution of correctly and incorrectly predicted residues in the two datasets using different cutoff values. Apparently, more residue positions are considered to be incorrect if a smaller cutoff (e.g. 2.0 Å) is used while a larger number of residues are considered correct if a large cutoff is used. The number of correct and incorrect residues was almost balanced at a cutoff of 4.0 and 5.0 Å. Comparing the L-E benchmark and the CASP9 datasets, both datasets showed essentially the same trend but the overall quality of models in the L-E dataset was better than those in the CASP9 sets.

## Results and Discussion

We start by discussing the size of cliques in protein structure graphs, which was found to have a large influence in the resulting quality assessment accuracy. Then, we will present the prediction results on the benchmark dataset. The accuracy of the method under different conditions will be thoroughly discussed. Finally, we compare the performance of GMQ with other existing methods on CASP protein structure models.

### Distribution of Clique Size

A unique aspect of GMQ is that the predicted qualities of neighboring residues are taken into account in predicting the quality of a residue (Eq. 4). Thus, the size of cliques in a structure model is an important factor that can affect to the accuracy of quality estimation.

**Figure 3:**
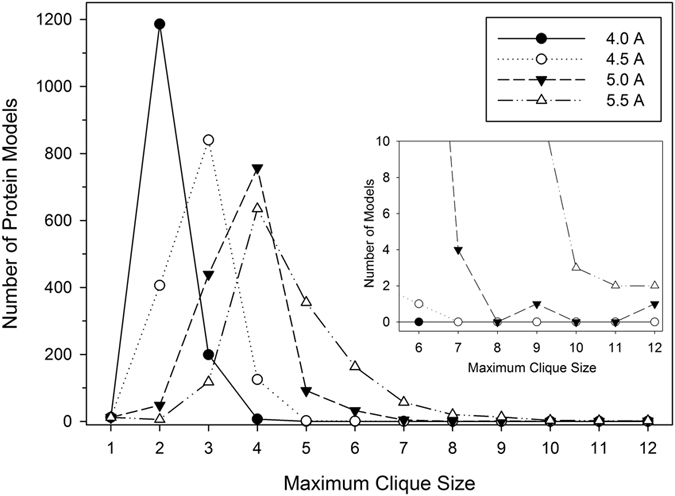
Distribution of the maximum clique size in structure model in the L-E benchmark set. Five different distance cutoff values, 4.0, 4.5, 5.0, and 5.5 Å, that define neighboring residues were used.

In Fig. 3, the distribution of maximum clique sizes in each structure model in the L-E benchmark dataset is shown. Larger cliques were constructed in models as the neighbor distance cutoff increased as indicated with the noticeable peak shift with larger cutoffs. The largest clique in the majority of structure models was 2 when the neighbor cutoff of 4.0 Å was used. The peak shifts to 3 with a 4.5 Å cutoff and further to 4 with 5.0 and 5.5 Å cutoff values. With the neighbor cutoff of 4.5 Å, no structure models had a clique of a size of 7 or larger. On the other hand, using a cutoff of 5.0 Å, six models had a clique larger than seven nodes, with one model with a clique of 12 nodes. With a 5.5 Å cutoff, all models but one had a clique of a size 3 or larger.

**Figure 4:**
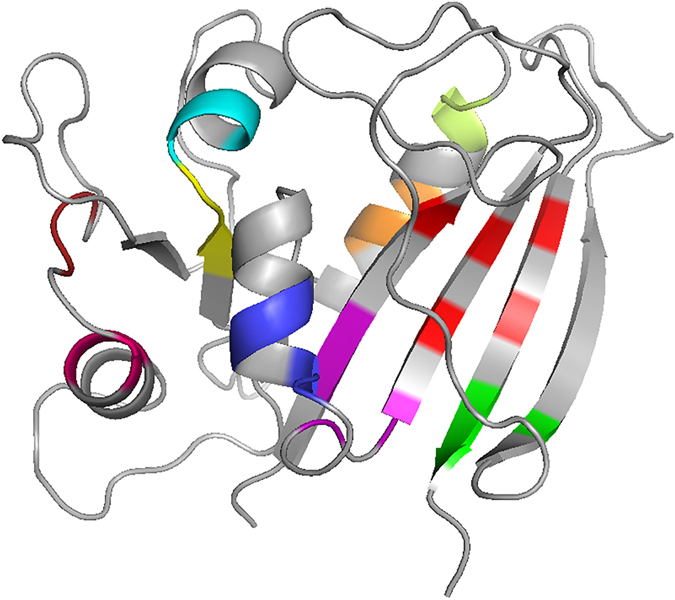
Examples of cliques constructed for a model. This is a model for dihydrofolate reductase (PDB ID: 1dr4; 186 residue long). A neighboring residue cutoff of 5.0 Å was used. Residues in the same color are included in the same clique.

Figure 4 shows examples of cliques in a structure model. Residues in the same color form a clique. Often cliques consist of residues that are distant in the sequence. As shown in the figure, it is typical that residues distant on the sequence but physically close in adjacent β-strands are included in the same clique (e.g. cliques in red, green, and magenta). Also contiguous residues in an α-helix tend to form a clique (e.g. residues in blue, orange, cyan). The colored residues are just several examples of the cliques in this protein model. In total, there are 71 cliques that have three residues or more including four cliques with five residues.

### Quality Prediction Results on the L-E dataset

**Figure 5:**
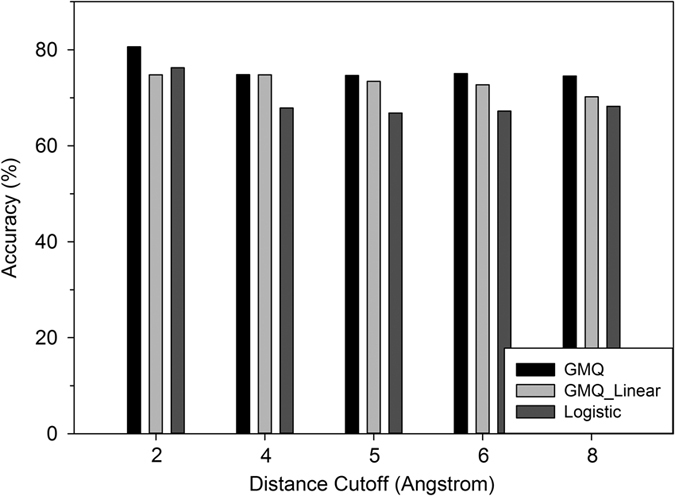
Results on benchmark dataset using CRF (GMQ), GMQ with a neighboring residue cutoff of 1.0 Å (Linear), and logistic regression. For GMQ, a neighboring residue cutoff of 4.5 Å was used to construct cliques. Distance cutoffs that define correctly/incorrectly predicted residues are shown on the X-axis while the Y-axis represents the accuracy, i.e. the fraction of residues whose label are correctly predicted.

In this section, we discuss the quality prediction results of GMQ on the L-E benchmark dataset. GMQ performs binary classification of residues in a model into correct and incorrect predictions. Correctly predicted residues are defined as those which are placed within a certain cutoff distance (2.0, 4.0, 5.0, 6.0, and 8.0 Å between corresponding Cα atoms) to the correct position. To compute the accuracy, the L-E benchmark dataset was split into five subsets, of which four subsets were used for training parameters of the CRF and the remaining subset was used for testing. This was repeated five times changing the testing set each time (i.e. a five-fold cross validation) and the average accuracy of the classification was reported in Fig. 5. Figure 5 shows the accuracy of GMQ (with the neighboring residue cutoff of 4.5 Å for connecting edges in a protein graph) using different Cα distance cutoff values. The performance of GMQ was compared with two baseline methods: One is GMQ using a small neighboring residue cutoff of 1.0 Å, much smaller than the average Cα – Cα distance (3.8 Å) in a protein structure, so that practically a clique does not have more than one residue (denoted as GMQ_linear), and logistic regression where a binary classification for each residue was performed by only using residue features. Comparison with logistic regression was motivated because our former QA method, Sub-AQUA^21^ is based on logistic regression. In addition to the 25 features, prediction performance with a five feature combination was also examined as described in the Residue Features section in Methods.

Figure 5 shows that GMQ consistently outperforms logistic regression. With GMQ_linear, GMQ showed better performance using four Cα distance cutoffs, 2.0, 5.0, 6.0, and 8.0 Å, and tied for 4.0 Å. These results indicate that in general neighborhood information is effective in improving prediction accuracy. We also examined the performance of GMQ with the five features (see the Method section). With the five feature combination, the prediction accuracy of GMQ dropped from 74.64% to 70.56% for a 5.0 Å Cα distance cutoff.

Next, we examined how the neighbor cutoff value, which defines the clique size, affects the accuracy. As the neighbor cutoff value is increased, more neighboring residues will be involved in predicting the quality of a target residue. Table 1 compares pairs of neighbor cutoff values by counting the number of structure models whose quality was predicted better by using larger cutoff values. Table 1 shows that more models are better predicted by using a larger neighbor cutoff value that makes larger cliques. For example, comparing neighbor cutoff of 4.5 and 4.0 Å, quality of 72.15% of the structure models were better predicted by using a 4.5 Å cutoff. This result is consistent with Fig. 5 where GMQ, which consider neighboring residues, was compared with GMQ with single feature combinations, and logistic regression, both of which do not take neighbor into account.

To understand the contribution of each feature to the prediction accuracy of GMQ, we deleted each of the 25 features individually and examined how much accuracy dropped from the result shown in Fig. 5, which was 74.64% (with a 5.0 Å Cα cutoff). Among the 25 features, seven features reduced the accuracy more than 0.25% when removed. They were mutation score (0.49%), gap ratio (0.42%), the length of the aligned regions of target protein to a template (0.36%), contact order (0.35%), Maxsub score predicted by ProQ (0.29%), local SPAD (0.30%), and log(local SPAD / SPAD + 1) (0.27%). The mutation score is the average BLOSUM45 score^45^ and the gap ratio is the fraction of gaps, both at the position of the residue in the multiple sequence alignment of the query protein^21^. Thus, in GMQ, the most influential features included four alignment-based local features and three global features (the aligned length, contact order, and the predicted MaxSub score).

The 25 features did not have strong correlation with each other, when Pearson’s correlation coefficient was concerned. Among all pairwise combinations within the nineteen global features and six local features, only one had a correlation coefficient greater than 0.9, the length of a structure model and the aligned length of a model to its template protein. Removing a redundant feature, the aligned length, reduced the overall accuracy from 74.64% to 74.28% (a reduction of 0.36%), as mentioned above. The next largest correlation was observed between the length of the model and the Verify3D score (0.863). Removing the length feature (thus the “redundancy” with the Verify3D and the aligned length were both dissolved) decreased the overall accuracy to 74.43% (a reduction of 0.21%). All the other pairs have correlation coefficients of less than 0.7.

**Figure 6:**
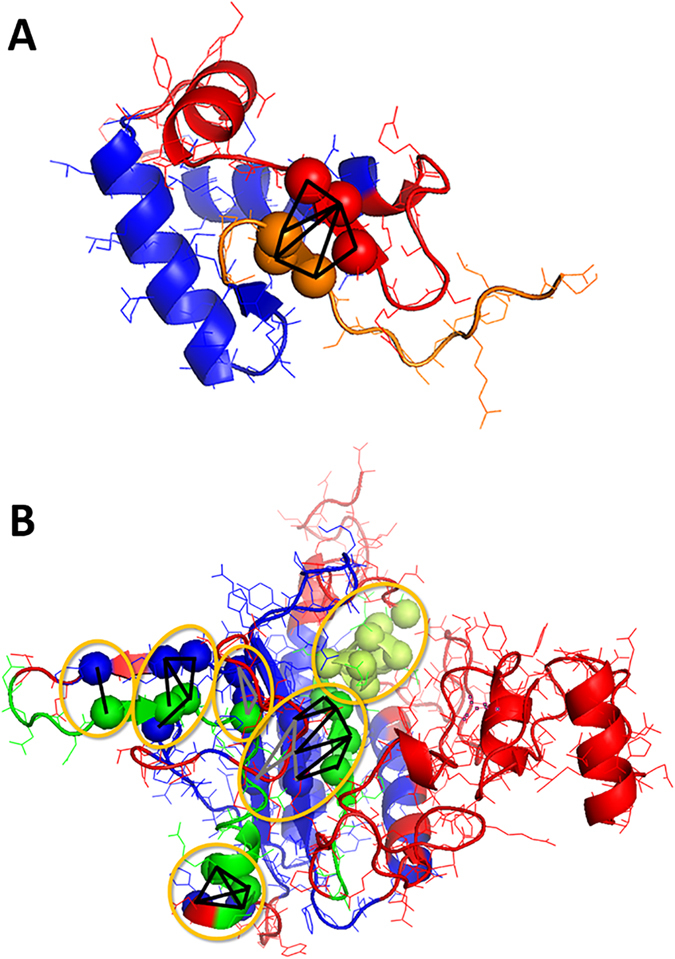
Examples of protein models whose quality was better predicted by using a larger neighbor cutoff value. Black lines show edges formed when a larger neighbor cutoff was used. (**A**) A model of globular domain of histone h5 (PDB: 1hstA, 74 residue long). Residues in blue are true positives. Red residues are true negatives. Orange residues were false positives by GMQ with a 4.0 Å neighbor cutoff but correctly classified as true negatives with a 5.0 Å cutoff. (**B**) A model of histidyl-tRNA (PDB ID: 1htt, 267 residue long). Blue and red are true positives and true negatives. Residues in green are false negatives when a neighbor cutoff of 4.0 Å was used, but GMQ with a 5.0 Å neighbor cutoff correctly classified them.

Figure 6 illustrates how quality prediction can improve by using larger cliques in GMQ. Quality assessments by GMQ with two neighbor cutoff values for cliques were compared. In the figures, residues in blue and red are true positives and true negatives, respectively, which were correctly and wrongly modelled and classified correctly as such by GMQ using either 4.0 and 5.0 Å neighbor cutoff values. Orange residues in Fig. 6A are false positives, which were modelled wrongly but predicted as a good quality by GMQ when a small neighbor cutoff, 4.0 Å, was used. In contrast, green residues in Fig. 6B are false negatives, which were modelled correctly but predicted to be low quality by GMQ with a 4.0 Å neighbor cutoff. However, GMQ made correct quality classification for the residues in orange and green by using a larger neighbor cutoff, 5.0 Å.

Orange residues in the first model (Fig. 6A) were misclassified by GMQ when a small neighbor cutoff of 4.0 Å was used. However, when the neighbor cutoff was increased to 5.0 Å making larger cliques, edges shown in black lines were formed, which bridged the orange residues with residues in red (true negatives) and helped GMQ to correctly classify them as incorrect. Similarly, in the second example (Fig. 6B), green residues were false negatives with a 4.0 Å neighbor cutoff. This misclassification was drastically improved by forming larger cliques with a 5.0 Å neighbor cutoff, which bridged the green residues with residues in blue, true positives. Local regions with cliques that showed drastic quality classification improvement by using a 5.0 Å cutoff are indicated by orange circles. At the upper right of Fig. 6B, a clique of many residues in yellow-green are those in an α-helix, which helped in propagating correct classification from the adjacent green residues.

### Using Different Edge Weights

Next, we investigated whether using different weights for different edge types improves the quality assessment accuracy. Particularly, we classified edges in a clique into five categories depending on secondary structures that are connected by edges, i.e. edges that connect residues in two helices (HH), residues in two β-strands (BB), a helix and a β-strand (HB), edges between a residue in coil and another one either in a helix or a β-strand (CH/CB), and edges between two coil regions (CC) (here by coil we mean any regions other than helices and β-strands). Changing edge weights by considering the secondary structures seemed intuitive because in general the core parts of a protein, which are dominated by helices or β-strands, are well conserved across related proteins and thus the quality of residues there may be closely related while residues in flexible loops (coils) are not easy to model. Applying different edge weight means, concretely, edges in cliques in the second term in Equation 4 now have different weights according to the secondary structure types rather than a uniform edge weight of 1.0. We performed a grid search for the five weight values from 0.5 to 1.0 for HH, BB, and HB while between 0.0 and 0.5 for HC/BC and CC with an interval of 0.1. The search range for weights for edges between coil regions is set lower because structures in coil regions are relatively less conserved across homologous proteins than helices and strand regions^53^,^54^.

**Figure 7:**
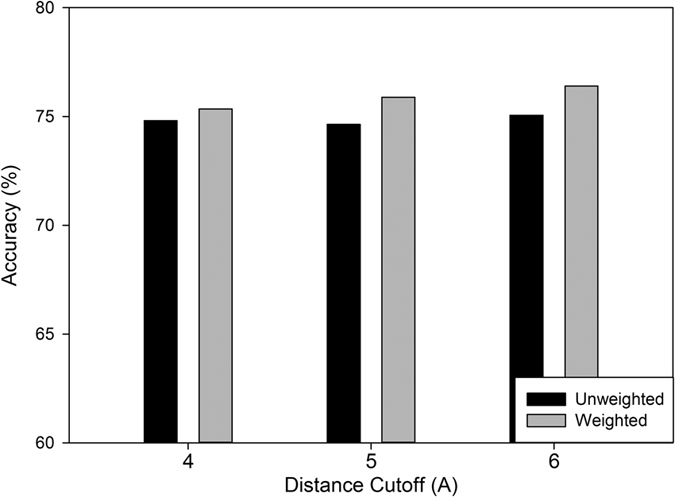
Accuracy with weighted and un-weighted edges in cliques. The neighbor cutoff was set to 4.5 Å. Results for 4.0, 5.0, and 6.0 Å were used as accuracy definition since they give good balance between the amount of correct and incorrect residues.

Figure 7 compares accuracy obtained by using different weights for different edge types and those using the same weights (1.0) for all five edge categories. The weight values obtained are 0.8–1.0 for HH, BB, HB, 0.1 for HC/BC, and 0.0 for CC (i.e. edges between residues in coils are effectively removed). Because the accuracies obtained with different combinations of weights among 0.8, 0.9, and 1.0 for HH, BB, and HB were very similar (the standard deviation was 0.37), we showed the average accuracy of the results. As shown in the figure, the edge-dependent weight scheme improved the accuracy. The improvement of the accuracy was 0.54, 1.25, and 1.34 for a Cα cutoff of 4.0, 5.0, and 6.0 Å, respectively, by using the edge-dependent weights.

**Figure 8:**
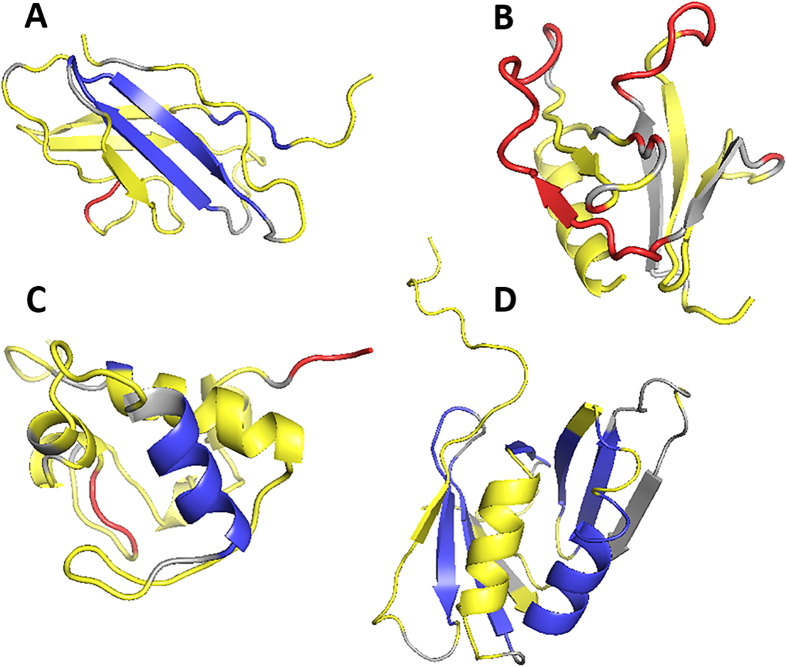
Examples of improved quality prediction by using edge weights. A distance cutoff of 4.0 Å was used to define accurately modeled residues while a neighboring residue cutoff of 5.0 Å was used to construct cliques. Residues in yellow are those whose quality (within/above 4.0 Å) were correctly predicted. Residues in blue/red are those which are miss-classified by GMQ with the equal edge weight scheme but correct classification was achieved by using different edge weights. Blue/red are correctly/incorrectly modeled residues, respectively. (**A**) A structure model of 1cfb (100 residue long). The quality prediction accuracy computed with the equal weights was 68.0% while it was improved to 89.0% when different edge weights was used. (**B**) A structure model for 1btn (106 residue long). The quality prediction accuracy with and without edge weights were 50.9% and 80.2%, respectively. (**C**) a model for 1cyj (89 residue long). The quality prediction accuracy with/without edge weights was 70.8/88.8%. (**D**) A structure model for 1plq (126 residue long). The quality prediction accuracy with/without edge weights were 41.3/76.2%.

Figure 8 illustrates the effect of using the edge-dependent weights for different edge types. Yellow regions are residues whose quality was correctly classified by GMQ using both edge weighting schemes. On the other hand, blue and red regions are where quality assessment improved by using edge-dependent weights over using the equal weight scheme. Blue indicates residues that are correctly modelled but predicted as incorrect by GMQ with equal edge weight (false negative). Red is the opposite: those are residues modelled incorrectly (over 4.0 Å), but classified as correct by GMQ with equal weight (false positive). For both red and blue residues, the inaccurate prediction was corrected by applying the edge-dependent weights.

As shown in Fig. 8A–C, the improvement of quality classification occurs not sporadically in a model but in a unit of large fragments of secondary structure elements. In Fig. 8A, a 16-residue long region with two β-strands (blue) in addition to two loop regions (one in red and another one in blue) were turned into correct classification. In Fig. 8B, two long eight- and 19-residue loops, which were originally misclassified as correct by GMQ with the equal weight scheme (red) were now classified as incorrect by using the edge-dependent weights. Figure 8C is an example that the quality of an α-helix (blue) was correctly classified with the edge-dependent weights. In addition, classification of two loops (red) is also fixed. The last example (Fig. 8D) shows a drastic improvement of quality assessment accuracy from 41.3% to 76.2% due to the correct classification of a large region of the β-sheet and an α-helix in the model (blue). In these examples, these secondary structure element level improvements are probably due to weakening connections between loops and helices/strands in cliques, effectively separating the quality prediction of loops, which are frequently modelled incorrectly, from helices and strands.

### Comparison with existing methods

Finally, we compared GMQ with existing single-model QA methods, which predict quality of models using only the model itself (without comparing the model with other models). For this comparison, we used prediction models from three recent CASP competitions, from CASP9^4^ (Tables 2, 3 and 4), CASP10^55^ (Tables 5, 6 and 7), and CASP11^56^ (Tables 8, 9 and 10). In CASP9, 21 groups participated in the local quality assessment. Of these, four groups were single-model methods, which we compared against GMQ. To compute the alignment features used in GMQ, we used a template protein specified by a model prediction group if the statement existed in the PDB file of the model. Otherwise, we ran BLAST^57^ against PDB to find a template. To obtain prediction to the CASP9 targets, GMQ was retrained using the L-E dataset after removal of proteins in the L-E dataset that have 30% or more sequence identity to any of the CASP9 targets. Similarly, on the CASP10 and CASP11 model datasets, we compared GMQ with three methods and five methods respectively, which are single-model QA methods. In the same way as the predictions were made for the CASP9 targets, GMQ was retrained with the L-E dataset after removal of homologous proteins to the CASP10 and the CASP11 targets from the dataset, respectively.

The performance of evaluated with the fraction of correctly classified residues at two cutoff values as was done in the official CASP evaluation CASP9^4^. In the performance comparison on the CASP9 dataset (Table 2), GMQ was ranked third among the five methods using either Cα cutoff value. According to paired t-tests (Table 3), the performance difference of the methods for the 5.0 Å Cα cutoff were not statistically significant when a p-value of 0.05 was considered. With the 3.8 Å Cα cutoff, all of the performance differences between methods were statistically significant except for GMQ and ProQ. All methods showed a higher prediction accuracy for template-based models (TBM) than free-modeling models (FM). The difference of prediction accuracy by GMQ between TBM and FM was relatively small, when compared with the other methods. In Table 3, we also evaluated the method’s performance with the Matthews correlation coefficient (MCC). The ranking of the methods was almost the same as in Table 2, except that GMQ was ranked second for the 5.0 Å Cα cutoff. On the CASP10 dataset (Table 5), GMQ was ranked second following MULTICOM-novel for the Cα 5.0 Å cutoff, and third using the 3.8 Å Cα cutoff. Table 6 shows statistical significance of the performance by every pair of methods. For the Cα 5.0 Å cutoff, the methods were not statistically distinguishable, while for the 3.8 Å Cα cutoff every consecutive method pair in the ranking was not distinguishable (Table 6). When evaluated with MCC, GMQ was ranked third for both Cα cutoff values (Table 7). When the CASP11 dataset was used (Tables 8, 9 and 10), GMQ was ranked fourth among the six methods in terms of the prediction accuracy (Table 8) and MCC (Table 10) for both Cα cutoff values. Thus, overall GMQ showed competitive performance in the CASP datasets for both cutoff values.

## Conclusion

Now that computational protein structure modeling is more routinely used for biological studies, quality assessment of models is becoming more important because it judges whether a model is accurate enough for a certain practical purpose. Among different types of model quality assessments, single-model local quality assessment is particularly useful, because many of practical uses of computational models concerns local structures, such as functional sites or molecular interaction sites in protein structures.

In this article we have reported a new approach named GMQ, which predicts local quality of a structure model by considering quality of neighboring residues. It was shown successfully that taking into account neighboring residues’ prediction indeed improves the quality assessment accuracy. CRF has been applied in protein structure prediction, which showed good performance^58^. However, in the existing structure prediction methods using CRF, a simple linear model was used. It is mainly because the 3D structure of a target protein is not determined during the computation process of structure prediction and thus cliques with distant residues cannot be formed. In contrast, here in GMQ, we use a general CRF model, which considers cliques that are connecting nodes (residues) distant to each other on the sequence. This work has shown that the neighbor information is very useful for quality assessment. Using the established framework of GMQ, exploring different features of residues is expected to further improve the accuracy of quality assessment.

## Additional Information

**How to cite this article:** Shin, W.-H. *et al*. Prediction of Local Quality of Protein Structure Models Considering Spatial Neighbors in Graphical Models. *Sci. Rep.* **7**, 40629; doi: 10.1038/srep40629 (2017).

**Publisher's note:** Springer Nature remains neutral with regard to jurisdictional claims in published maps and institutional affiliations.

## Figures and Tables

**Table 1:** Comparison of neighbor cutoff values.

		Cutoff values to be compared against (Å)		
Neighbor cutoff (Å)		4.0	4.5	5.0
	4.5	72.15		
	5.0	64.50	67.82	
	5.5	63.56	65.15	71.28

For each protein models in the benchmark dataset, the fraction of residues with correctly classified quality were compared. GMQ with the 25 feature combinations was used. The numbers shown are the percentage (%) of models whose quality prediction accuracy was better or equal by a larger neighbor cutoff value (left column). The Cα distance cutoff was set to 5.0 Å as the numbers of correct/incorrect residues are balanced.

**Table 2:** Performance comparison with other methods participated in CASP9.

Method	Cα 5.0 Å			Cα 3.8 Å		
	All	TBM	FM	All	TBM	FM
ProQ2	0.777	0.805	0.652	0.779	0.785	0.754
QMeandist	0.704	0.768	0.431	0.734	0.748	0.676
GMQ	0.701	0.720	0.621	0.657	0.663	0.632
ProQ	0.687	0.771	0.317	0.649	0.724	0.318
DistillNNIPF	0.669	0.761	0.267	0.619	0.706	0.241

Fraction of correctly classified residues in the dataset are reported using two cutoff values, 5.0 and 3.8 Å between Cα atoms. For GMQ, the edge-dependent weight scheme was used. The All columns show results on all the 115 targets; TBM columns show results on 93 template-based targets while FM columns show results for 22 Free modeling targets.

**Table 3:** Statistical significance (p-value) of performance difference of the method pairs on the CASP9 dataset.

	QMeandist	GMQ	ProQ	DistillNNIPF
5.0 Å				
ProQ2	<0.05	<0.05	<0.05	<0.05
QMeandist		0.140	<0.05	<0.05
GMQ			0.377	<0.05
ProQ				<0.05
3.8 Å				
PROQ2	<0.05	<0.05	<0.05	<0.05
QMeandist		<0.05	<0.05	<0.05
GMQ			0.493	<0.05
ProQ				<0.05

Paired t-test was performed for each pair of methods.

**Table 4:** Matthews correlation coefficient for the CASP9 dataset.

Method	Cα 5.0 Å			Cα 3.8 Å		
	All	TBM	FM	All	TBM	FM
ProQ2	0.485	0.396	0.382	0.534	0.455	0.434
QMeandist	0.298	0.242	0.215	0.446	0.366	0.397
GMQ	0.322	0.271	0.252	0.281	0.237	0.194
ProQ	0.192	0.194	0.124	0.223	0.223	0.146
DistillNNIPF	N/A					

The coefficient for DistillNNIPF was not computed because it predicted all residues are within 3.8 Å and thus both true negatives and false negatives were 0, which made the denominator 0^4^.

**Table 5:** Performance comparison with other methods participated in CASP10.

Method	Cα 5.0 Å			Cα 3.8 Å		
	All	TBM	FM	All	TBM	FM
MULTICOM-novel	0.701	0.692	0.730	0.593	0.532	0.781
GMQ	0.700	0.722	0.632	0.671	0.681	0.639
ProQ2	0.698	0.741	0.560	0.708	0.722	0.664
MULTICOM-cluster	0.696	0.718	0.630	0.684	0.671	0.724

The All columns show results on all the 90 targets; TBM columns show results on 75 template-based targets while FM columns show results for 15 Free modeling targets.

**Table 6:** Statistical significance (p-value) of performance difference of the method pairs on the CASP10 dataset.

	GMQ	ProQ2	MULTICOM-cluster
5.0 Å			
MULTICOM-novel	0.226	0.158	0.108
GMQ		0.251	0.386
ProQ2			0.308
	MULTICOM-cluster	GMQ	MULTICOM-novel
3.8 Å			
PROQ2	0.117	<0.05	<0.05
MULTICOM-cluster		0.123	<0.05
GMQ			0.493

Paired t-test was performed for each pair of methods.

**Table 7:** Matthews correlation coefficient on the CASP10 dataset.

Method	Cα 5.0 Å			Cα 3.8 Å		
	All	TBM	FM	All	TBM	FM
MULTICOM-novel	0.404	0.364	0.367	0.243	0.234	0.188
GMQ	0.381	0.362	0.280	0.349	0.338	0.275
ProQ2	0.413	0.389	0.326	0.469	0.439	0.383
MULTICOM-cluster	0.375	0.349	0.308	0.368	0.330	0.355

**Table 8:** Performance comparison with other methods participated in CASP11.

Method	Cα 5.0 Å			Cα 3.8 Å		
	All	TBM	FM	All	TBM	FM
Wang_deep_3	0.709	0.722	0.676	0.705	0.685	0.756
ProQ2	0.666	0.733	0.493	0.686	0.699	0.655
ProQ2_refine	0.662	0.732	0.482	0.683	0.697	0.646
GMQ	0.655	0.705	0.524	0.633	0.651	0.587
MULTICOM-novel	0.548	0.669	0.236	0.562	0.623	0.405
MULTICOM-cluster	0.529	0.654	0.208	0.478	0.565	0.243

The All columns show results on all the 68 targets; TBM columns show results on 38 template-based targets while FM columns show results for 30 Free modeling targets.

**Table 9:** Statistical significance (p-value) of performance difference of the method pairs on the CASP 11 dataset.

	ProQ2	ProQ2_refine	GMQ	MULTICOM-novel	MULTICOM-cluster
Wang_deep_3	0.055	<0.05	<0.05	<0.05	<0.05
ProQ2		<0.05	<0.05	<0.05	<0.05
ProQ2_refine			0.0839	<0.05	<0.05
GMQ				<0.05	<0.05
MULTICOM-novel					<0.05
3.8 Å					
Wang_deep_3	0.300	0.237	<0.05	<0.05	<0.05
ProQ2		<0.05	<0.05	<0.05	<0.05
ProQ2_refine			<0.05	<0.05	<0.05
GMQ				<0.05	<0.05
MULTICOM-novel					<0.05

Paired t-test was performed for each pair of methods.

**Table 10:** Matthews correlation coefficient on the CASP11 dataset.

Method	Cα 5.0 Å			Cα 3.8 Å		
	All	TBM	FM	All	TBM	FM
Wang_deep_3	0.419	0.376	0.207	0.408	0.361	0.212
ProQ2	0.388	0.377	0.181	0.447	0.410	0.241
ProQ2_refine	0.382	0.374	0.179	0.444	0.407	0.238
GMQ	0.318	0.322	0.171	0.298	0.287	0.158
MULTICOM-novel	0.146	0.157	0.076	0.236	0.233	0.130
MULTICOM-cluster	0.069	0.068	0.006	0.099	0.083	0.006
